# Overexpression of CBX3 in Pancreatic Adenocarcinoma Promotes Cell Cycle Transition-Associated Tumor Progression

**DOI:** 10.3390/ijms19061768

**Published:** 2018-06-14

**Authors:** Lian-Yu Chen, Chien-Shan Cheng, Chao Qu, Peng Wang, Hao Chen, Zhi-Qiang Meng, Zhen Chen

**Affiliations:** 1 Department of Oncology, Shanghai Medical College, Fudan University, Shanghai 200032, China; cly@shca.org.cn (L.-Y.C.); natcheng@connect.hku.hk (C.-S.C.); quchaolove@163.com (C.Q.); wangp413@163.com (P.W.); chengkll@126.com (H.C.); mengzhq@yeah.net (Z.-Q.M.); 2 Department of Integrative Oncology, Fudan University Shanghai Cancer Center, Shanghai 200032, China

**Keywords:** CBX3, pancreatic adenocarcinoma, cell cycle regulation, CDK1, tumor progression

## Abstract

Background: Previous studies showed that Chromobox protein homolog 3 (CBX3) was overexpressed in several types of human cancers, however its pattern and role in pancreatic adenocarcinoma (PAAD) has not yet been understood. The aim of this study was to identify the expression and function of CBX3 in PAAD. Methods: Data of transcriptomic and protein expression of CBX3 in PAAD were collected from different databases and analyzed. The in vitro and in vivo role of CBX3 in PAAD was examined. Results: CBX3 was overexpressed in human PAAD tissues, which was associated with poor prognosis of overall and disease-free survival of the patients. Overexpression of CBX3 induced the in vitro proliferation, anchorage-free growth, migration and invasion of the PAAD cells, and led to in vivo growth of orthotoptic PAAD tumors in mice. GO and KEGG pathway analysis, as well as experimental observation showed that CBX3 may be associated with cell cycle transition of PAAD cells, and cyclin-dependent kinase 1 (CDK1) and proliferating cell nuclear antigen (PCNA) may mediate the tumor-promoting action of CBX3. CDK1 knockdown attenuated the cell cycle transition, proliferation and invasion of CBX3-overexpressing PAAD cells. Conclusion: Our findings suggest the tumor-promoting role of CBX3 in PAAD to be targeted by novel therapeutic strategies.

## 1. Introduction

Pancreatic cancer is one of the most malignant human cancers all over the world [1]. The most common type of human pancreatic cancers is pancreatic adenocarcinoma (PAAD), which accounts for more than 85% cases of pancreatic cancers [2]. Responsible for around 7% cancer-associated death in both men and women, the morbidity and mortality of PAAD is still rising, especially in developed countries [3]. By 2030, pancreatic cancer has been predicted to be the second leading causes of cancer-related death in the United States [4] and Germany [5]. Mechanisms underlying the tumorigenesis of PAAD remain to be explored, however, genetic factors as well as medical history of chronic pancreatitis, diabetes and obesity are considered to be the risk factors of PAAD [6]. Life styles such as alcohol consumption and smoking are associated with PAAD incidence [7]. Early diagnosis of PAAD seems difficult, as its symptoms at the early stage are not very observable and distinctive. Diagnosis often at late stage has made prognosis of PAAD very poor as cancer cells may have spread to other parts of the body [8]. Surgery is still the major management in PAAD with intension of cure; unfortunately, only around 20% of cases are suitable for surgical operation [9]. Non-invasive treatments such as chemotherapy and radiotherapy gain very poor outcome as PAAD cells are highly resistant [10]. In this case, identification of the pathological basis as well as molecular target of PAAD is quite necessary and emerging.

The Chromobox protein homolog 3 (CBX3) encodes a heterochromatin protein 1γ (HP1γ) in human cells that binds DNA as a component of heterochromatin. Some recent studies have identified the expression and role of CBX3 in different type of human cancers. In prostate cancer, CBX3 expression was elevated, and might be an independent factor to predict the biochemical recurrence of prostate cancer after radical prostatectomy [11]. CBX3 expression was increased in human colorectal cancer and promoted in vitro and in vivo proliferation of the tumor cells, which was associated with its regulation on CDKN1A in colorectal cancer cells [12]. Further study demonstrated that CBX3 can suppress the transcription of CDK6 and p21, the negative cell cycle regulators, and promote proliferation of colorectal cancer cells [13]. Similar observation was obtained in tongue squamous cell carcinoma, in which overexpression of CBX3 inhibited p21 and promoted G1/S cell cycle transition [14]. In addition, CBX3 was found overexpressed in breast cancer and non-small cell lung cancer tissues, and its expression may predict the poor prognosis of patients with these cancers [15,16]. A recent study showed that CBX3 deficiency may mitigate the tumor burden in mice with treatment of tumor-killing CD8+ cells, further suggesting its rational role as a therapeutic target in solid tumor treatment [17]. These increasing lines of evidence have suggested the important role of CBX3 in the progression and treatment of human cancers, however, the expression and mechanism of action of CBX3 in pancreatic cancer has not yet been fully understood.

In this study, we aimed to explore the expression and function of CBX3 in pancreatic cancer. We extracted transcriptomic data of human PAAD tissue from GEO database and compared the expression of CBX3 in PAAD and non-tumor tissues from two datasets. Overexpression of CBX3 in PAAD cells was achieved by Crispr-cas9 activation, and in vitro and in vivo tumor cell growth and invasion was measured. Correlation and gene ontology analyses were performed to understand the mechanism network of CBX3 that may be involved.

## 2. Results

### 2.1. CBX3 Was Overexpressed in PAAD and Predicted Poor Prognosis of Patients

**Figure 1:**
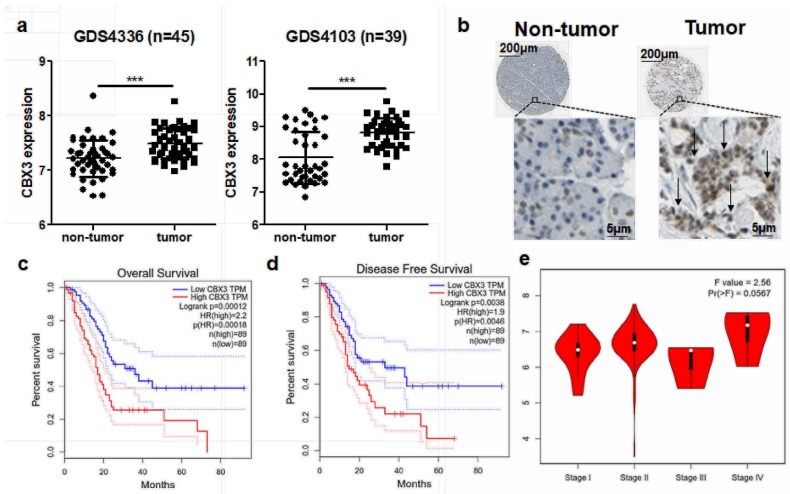
CBX3 was overexpressed in PAAD and predicted poor prognosis. (**a**) Data of CBX3 expression in human PAAD and non-tumor tissues were extracted from GEO dataset GDS4336 and GDS4103. It was shown that CBX3 was highly expressed in PAAD tissue. *** *p* < 0.001 when comparison was made between groups; (**b**) Protein expression of CBX3 was accessed from Human Protein Atlas project. Expression of HP1γ in human PAAD slides was significantly increased, as evidence of positive staining of the protein (brown dots). The black arrows showed cells with strong expression of HP1γ; (**c**) Data of expression of CBX3 and survival time of corresponding patients were extracted from TCGA database. KM plots showed that patients with CBX3 expression higher than median level had shorter overall survival. The area between the upper and lower blue/red dash lines indicated areas within 95% confident intervals (CIs); (**d**) Data of expression of CBX3 and survival time of corresponding patients were extracted from TCGA database. KM plots showed that patients with CBX3 expression higher than median level had shorter disease-free survival. The area between the upper and lower blue/red dash lines indicated areas within 95% CIs; (**e**) Data were collected from Gepia database. The results showed that CBX3 was increase during the disease progression of PAAD.

The role of CBX3 has been studied in various types of human cancers, such as breast cancer, non-small cell lung cancer, colorectal carcinoma and prostate cancer. However, its role and significance in PAAD is not yet understood. To identify the clinical significance of CBX3 in PAAD, we first extracted expression data of CBX3 from two released GEO microarray datasets examining human PAAD and non-tumor pancreas transcriptomic profiles. In both datasets, GDS4336 and GDS4103, expression of CBX3 was found significantly elevated in PAAD tissues compared with non-tumor pancreas (Figure 1a). This was further supported by the expression of CBX3-encoding protein, Heterochromatin protein 1γ (HP1γ). As examined by Human Protein Atlas project, HP1γ was highly expressed in PAAD tissues compared with non-tumor pancreas (Figure 1b). These data suggested the differential expression of CBX3 gene in PAAD. To further understand the prognosis value of CBX3 overexpression in PAAD, we collected patient survival information from TCGA database. The result showed that high expression of CBX3 in PAAD tissues predicted a poor prognosis of cancer patients in overall survival (Figure 1c). Its expression was also inversely associated with the disease-free survival of PAAD patients who received primary treatment (Figure 1d). In addition, expression of CBX3 is slightly associated with stage progress of PAAD, in which late stage patients seemed to have higher expression pattern of CBX3 in tumor tissues (Figure 1e). These findings revealed that CBX3 may be correlated as a tumor-promoting factor with PAAD progression.

### 2.2. CBX3 Promoted the In Vitro Proliferation and Invasiveness of PAAD Cells

**Figure 2:**
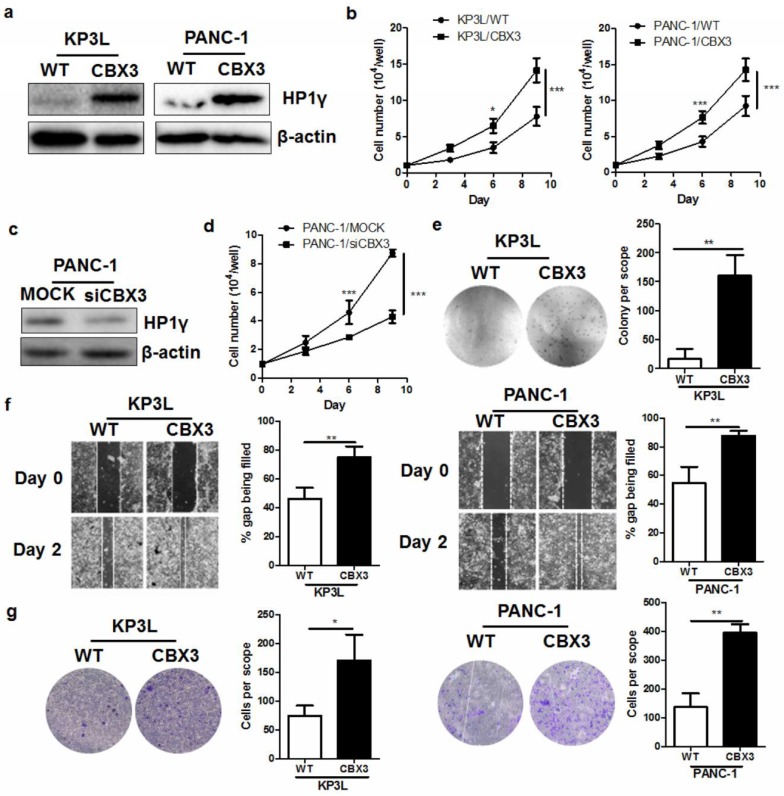
CBX3 overexpression increased in vitro proliferation and invasion of PAAD cells. (**a**) Expression of HP1γ was increased in CBX3-overexpressing KP3L and PANC-1 cells; (**b**) KP3L and PANC-1 cells with or without CBX3 overexpression were seeded at the density of 10^4^/well and allowed proliferation. Cell number was counted at Day 3, 6 and 9 after seeding. Overexpression of CBX3 significantly accelerated the proliferation of cells; (**c**) Expression of HP1γ was knocked down in PANC-1 cells with CBX3 siRNA; (**d**) Knockdown of CBX3 in PANC-1 cells reduced the proliferation rate of the cells; (**e**) Overexpression of CBX3 maintain the anchorage-free growth of KP3L and PANC-1 cells; (**f**) Overexpression of CBX3 induced the migration of KP3L and PANC-1 cells towards the center of the wound; (**g**) Overexpression of CBX3 promoted KP3L and PANC-1 cell invasion through extracellular matrix. In all panels, * *p* < 0.05, ** *p* < 0.01 and *** *p* < 0.001 when comparison was made between groups.

To understand if CBX3 can promote tumor cell proliferation and invasion in PAAD, we introduced Crispr-cas9 activation plasmid to induce overexpression of CBX3 in PAAD cell line KP3L and PANC-1. Stable expression of CBX3 Crispr-cas9 activation plasmid increased the HP1γ protein expression, as proved by Immunoblotting (Figure 2a). Cell count on the proliferation of KP3L and PANC-1 cells expressing scramble vector (KP3L/WT and PANC-1/WT. respectively) and KP3L and PANC-1 cells expressing CBX3-activation plasmid (KP3L/CBX3 and PANC-1/WT, respectively) showed that induced expression of CBX3 can accelerate cell proliferation (Figure 2b). To further confirm the role of CBX3, we then knockdown the expression of CBX3 in PANC-1 cells using RNA interference (Figure 2c). Knockdown of CBX3 in PANC-1 cells reduced its proliferation, further proving that CBX3 play a promoting role in PAAD cell proliferation (Figure 2d). Soft agar assay examining the colongenic property of anchorage-independent tumor cells revealed that CBX3 overexpression increased the anchorage-free growth of PAAD cells (Figure 2e). These findings have suggested that CBX3 may play an important role in promoting tumor cell proliferation in PAAD. In addition, overexpression of CBX3 increased the movement of KP3L and PANC-1 cells towards the wound center in wound healing assay (Figure 2f), as well as promoted their invasion through extracellular matrix (Figure 2g), suggesting that CBX3 overexpression can result in increasing aggressiveness of PAAD cells.

### 2.3. CBX3 Overexpression Accelerated In Vivo Tumor Progression of PAAD

**Figure 3:**
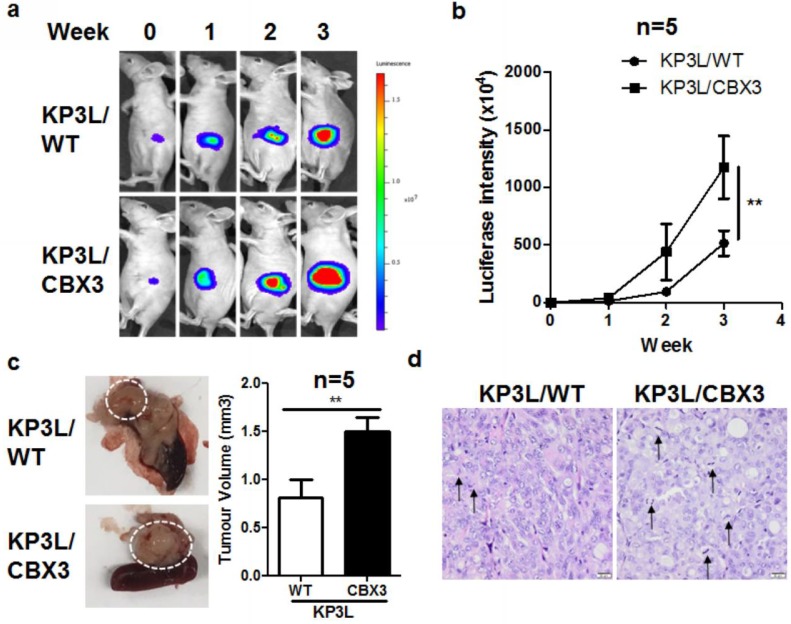
CBX3 overexpression promoted in vivo tumor progression of PAAD cells. (**a**) Luciferase-tagged KP3L cells were implanted to the pancreas of nude mice (*n* = 5). Luciferase was checked once per week for four weeks; (**b**) Increased luciferase intensity could be observed throughout the experiment, and CBX3 overexpressing KP3L cells showed a more rapid increase of luciferase intensity; (**c**) Size of pancreatic tumor formed by CBX3-overexpressing KP3L cells at the end of experiment was larger; (**d**) H&E staining revealed that more cells undergoing mitosis, indicating accelerating proliferation of the tumor cells. The black arrows highlighted cells undergoing rapid proliferation. In all panels, ** *p* < 0.01 when comparison was made between groups.

To further understand the in vivo oncogenic role of CBX3 overexpression, we established the PAAD orthotopic implantation model in athymic nude mice. KP3L cells expressing luciferase reporter were injected into the pancreas of nude mice to allow non-invasive live imaging of tumor growth. It was observed that overexpression of CBX3 in KP3L cells promoted the orthotopic growth of tumor cells by time (Figure 3a), as evidenced by the quantification of luciferin signal intensity (Figure 3b). At the end of the study, mice were sacrificed and pancreas was dissected out. Observation on the size of implanted tumor on pancreas proved that CBX3 overexpression can significantly accelerate tumor growth (Figure 3c). To further understand the action of CBX3, we performed histological analysis on the Haemotoxylin and Eosin (H&E) stained slides of tumor tissue. CBX-overexpressing tumor was found to contain more cell undergoing mitotic proliferation, as evidenced by condense chromosome presentation (Figure 3d). This further demonstrated that CBX3 overexpression can increase tumor growth. Taken together, both in vitro and in vivo evidence suggested that CBX3 play a tumor-promoting role in PAAD.

### 2.4. CBX3 May Be Involved in the Regulation of Cell Cycle Progression of PAAD

**Figure 4:**
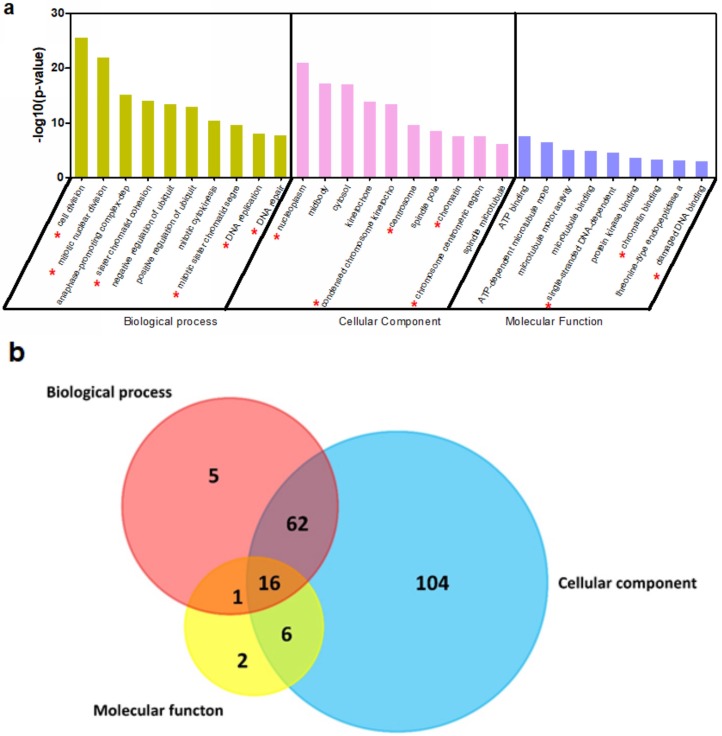

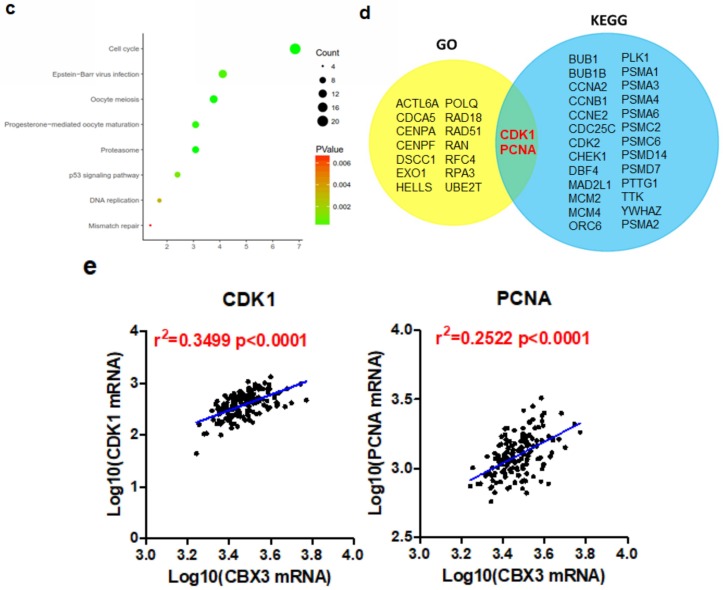
CBX3 may regulate the cell cycle transition of PAAD cells. (**a**) Correlated genes were retrieved from TCGA data and processed with GO annotation. Biological process (BP), Molecular function (MF) and cell component (CC) of the correlated genes were predicted. It was shown that CBX3-correlated genes were mostly related to cell cycle regulation and DNA replication. Items tagged with red asterisk were related to DNA replication and cell cycle regulation; (**b**) Common genes among cell cycle regulations from BP, MF and CC annotation was extracted by Venn diagram. Sixteen genes were shared in common; (**c**) KEGG pathway analysis with CBX3-correlated genes. It was consistently shown that cell cycle regulation may be involved; (**d**) Common genes between GO annotation and KEGG analysis revealed that CDK1 and PCNA may be the core element in the regulation of cell cycle by CBX3; (**e**) Expression of CBX3, CDK1 and PCNA in PAAD tissue was extracted, and correlation was analyzed by linear regression. It was shown that CDK1 and PCNA expression in PAAD tissue was positively correlated to CBX3 expression.

CBX3 has been observed to mediate various cellular action in different cancer cells. To predict the mechanism underlying its tumor-promoting activity in PAAD, we first extracted genes that were predicted to have median-to-strong correlations with CBX3 in PAAD. Only genes that had a Pearson’s correlation >0.4 or <−0.4, and spearman’s correlation >0.4 or <−0.4, were included. Overall, 241 genes were shown to be positively correlated with CBX3 while 55 had inverse correlation. These genes were put into David analysis to annotate the possible biological process (BP), cellular component (CC) and molecular function (MF) involved (Figure 4a). Interestingly, it was found that a majority of gene ontology (GO) items was related to the DNA replication and cell cycle regulation. Genes related to these activities were selected and we found that 16 genes were shared in common among the three GO enrichments, indicating the core role of these genes (Figure 4b). KEGG pathways that may be involved were also annotated, and the result, which was similar to the GO analysis, showed that CBX3 may be associated with genes in the pathways regulating cell cycle (Figure 4c). To further find the key genes that are associated with CBX3’s action, we overlapped the common genes from GO analysis and those from KEGG analysis. Two genes, named CDK1 and PCNA, were potentially core elements in both analyses (Figure 4d). To understand the clinical significance of the correlation, we analyzed the expression pattern of CBX3 in association with either CDK1 or PCNA in PAAD tissues. It was confirmed that, in PAAD tissues, expression of CDK1 and PCNA were positively correlated to CBX3, further suggesting that the regulation of CBX3 on PAAD progression may be related to CDK1 and PCNA.

### 2.5. CBX3 May Be Involved in the Regulation of G2/M Transition of PAAD Cells

**Figure 5:**
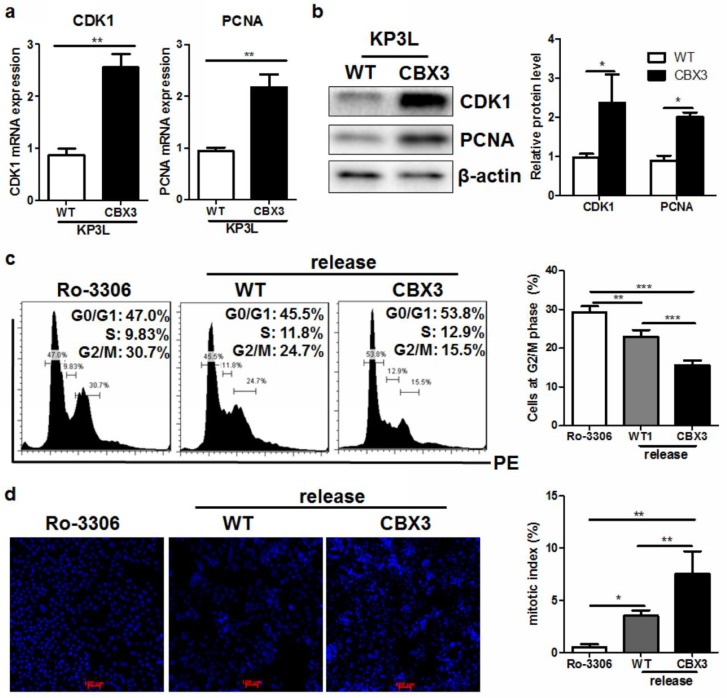
CBX3 overexpression promoted G2/M cell cycle transition. (**a**) mRNA expression of CDK1 and PCNA was increased in CBX3-overexpressing KP3L cells; (**b**) Protein expression of CDK1 and PCNA was increased in CBX3-overexpressing KP3L cells; (**c**) KP3L cells were arrested at G2/M phase by treatment of 5 μM Ro-3306 for 12 h. Medium was then replaced with fresh full medium and allow cell cycle progression for another 12 h. It was found that treatment of Ro-3306 accumulated cells at G2/M phase, and after release CBX3-overexpressing cells showed a more rapid transition of cell cycle as evidence of low G2/M portion; (**d**) KP3L cells were fixed and stained with DAPI. Cells with condensed nuclear were counted as mitotic cells under fluorescence microscope. Percentage of mitotic cells out of total viable cells was calculated as mitotic index. CBX3-overexpressing cells showed higher mitotic index than WT cells. In all panels, * *p* < 0.05, ** *p* < 0.01 and *** *p* < 0.001 when comparison was made between groups.

The Cyclin dependent kinase 1 (CDK1) is an important regulator of cell cycle transition, where it forms a regulatory element with Cyclin B1 during the progression from G2 phase to M phase [18]. CDK1 overexpression has been documented in various kinds of human cancers, and was found to be correlated with rapid progression of tumor [19,20,21,22]. The proliferating cell nuclear antigen (PCNA) is a facilitative element during DNA synthesis during the proliferation and spreading of tumor cells [23]. To further understand of role and regulation of CBX3, we first examined the expression of CDK1 and PCNA in CBX3-overexpressing PAAD cells. It was observed that upon induction of CBX3, CDK1 and PCNA were elevated (Figure 5a), which was consistent with their clinical patterns. The increased mRNA of CDK1 and PCNA in CBX3-overexpressing PAAD cells resulted in an induction of both proteins (Figure 5b). To further identify the functional role of CBX3 in cell cycle progression, especially CDK1-associated G2/M transition, we performed cell cycle analysis on KP3L/CBX3 cells. Cells were pretreated with Ro-3306, a CDK1 inhibitor. Treatment of Ro-3306 resulted in halt of cell cycle at G2 phase (Figure 5c). Ro-3306 was then withdrawn to release the cell cycle. It was found that CBX3 overexpression can proceed a higher transition from G2 phase after Ro-3306 withdrawal. Compared with KP3L/WT cells, KP3L/CBX3 had lower portion of cells staying at G2 phase (Figure 5c). Furthermore, CBX3 overexpression increase the mitotic index of KP3L cells after release, indicating a faster dividing and expansion of CBX3-overexpressing KP3L cells. These finding suggested that CBX3 may mediate a CDK1-associated cell cycle progression in PAAD cells.

### 2.6. The Regulation of Cell Cycle Transition by CBX3 Is Mediated by CDK1

**Figure 6:**
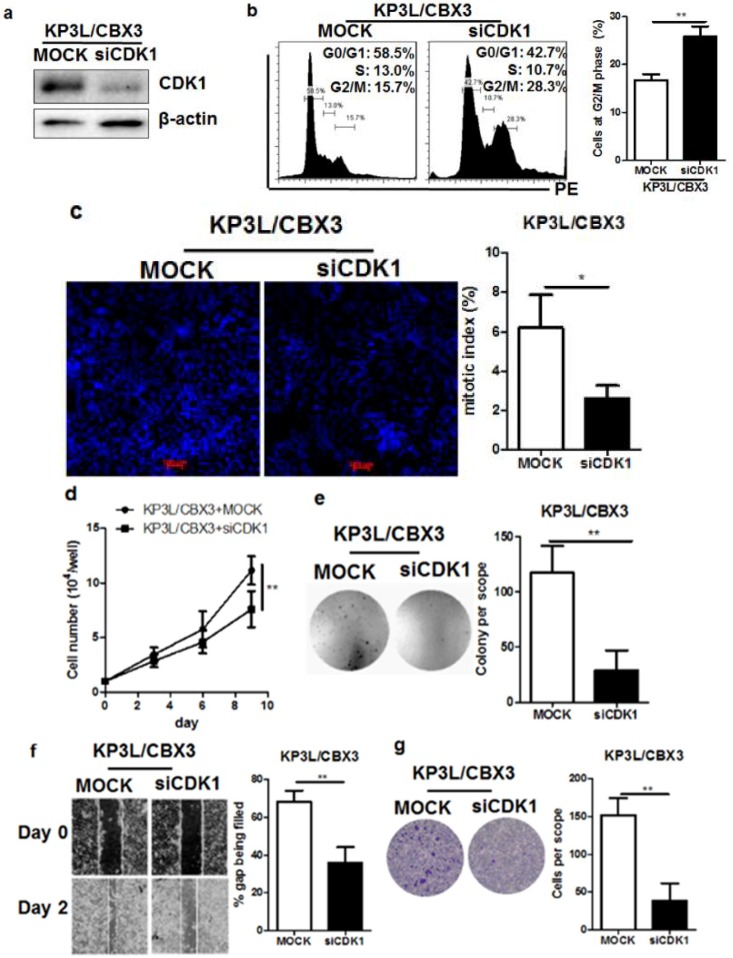
CDK1 mediated the tumor-promoting effect of CBX3 in PAAD cells. (**a**) Expression of CDK1 in CBX3-overexpressing cells was knockdown by siRNA and validated by Immunoblotting. (**b**) Knockdown of CDK1 increased G2/M accumulation of CBX3-overexpressing KP3L cells. Knockdown of CDK1 reduced cell: (**c**) mitotic index; (**d**) proliferation; (**e**) anchorage-free growth; (**f**) migration; and (**g**) invasion of the CBX3-overexpressing KP3L cells. In all panels, * *p* < 0.05 and ** *p* < 0.01 when comparison was made between groups.

To further examine if the regulation of CBX3 on cell cycle progression was mediated by the presence of CDK1, we used RNA interference to transiently knockdown the CDK1 in KP3L/CBX3 cells. Transfection of shRNA against CDK1 successfully reduced CDK1 expression in KP3L/CBX3 cells (Figure 6a). Knockdown of CDK1 in CBX3-overexpressing cells arrested cell cycle at G2/M phase, as evidenced by more cells distributed at G2/M checkpoint (Figure 6b). Knockdown of CDK1 reduced the mitotic index of CBX3-overexpressing cells (Figure 6c). This resulted in reduced in vitro proliferation of CBX3-overexpressing cells (Figure 6d), as well as the anchorage-free growth of tumor cells (Figure 6e). Migration and invasion of CBX3-overexpressing PAAD cells was also suppressed by CDK1 knockdown (Figure 6f,g). These findings suggested that the tumor-promoting effect of CBX3 may be mediated by CDK1 in PAAD cells.

## 3. Discussion

The human CBX3 gene encodes a protein named HP1γ that belongs to heterochromatin protein 1 (HP1) family. Proteins in this family were originally identified to lie at the core of the most conserved form of heterochromatin, which contains gene-poor and transcriptionally repressed regions [24]. In this case, HP1 proteins were initially regarded to function for heterochromatin spread and chromatin condensation [25]. However, more and more recent studies have revealed their new role as an epigenetic factor in transcription activation and elongation, sister chromatid cohesion, chromosome segregation, telomere maintenance, DNA repair, and RNA splicing [26]. The three paralogs of HP1 proteins, HP1α, HP1β and HP1γ, may have distinctive localizations and functions during gene regulation. In cancers, expression of HP1 proteins may be differential, which suggests their different roles in cancer progression by mediating regulation on gene expression [27]. The expression of proteins in HP1 family was differential in cancers, in which HP1α and HP1β was found down-regulated and HP1γ was overexpressed. While the expression and function of HP1γ has not yet been studied, there were a few of previous discussion and observation on the dysregulation of HP1α and HP1β in cancers. Early assumption on the de-regulation of HP1 in cancers referred to the observation that mutation might occur on the gene regions encoding these proteins [27]. However, recent studies showed that dysregulation of HP1 proteins can be mutation-independent. In breast cancer, transcription factor Yin Yang 1(YY1) was suggested to be responsible for the reduced expression of HP1α [28]. In ovarian cancer, expression of HP1α was repressed by Bromodomain-containing protein 4 (BRD4), which defects DNA damage response to facilitate tumorigenesis [29]. Defective type III secretion mxiD Shigella flexneri strain can lead to more phosphorylation of HP1γ protein and regulated its activity, which might be related to the infections-controlled HP1γ protein. Thus far, there were no clear evidence indicated how HP1 proteins were dysregulated in pancreatic cancers, as the role of HP1 proteins in PAAD is still under investigation. Mechanisms underlying dysregulation of HP1 proteins in PAAD shall be studied in detail in the soon future. In our study, we found that overexpression of CBX3 gene resulted in HP1γ production, and up-regulated the mRNA and protein levels of CDK1 and PCNA, suggesting a transcriptional activation mechanism may be involved in the HP1γ-mediated regulation of CDK1 and PCNA expression in PAAD. Some previous studies have shown that expression of HP1γ may transcriptionally repress the expression of tumor suppressive p21 gene through regulating the methylation of histone H3K9 on its promoter [12]. HP1γ was therefore considered to be associated with histone methylation and disassembly of transcription factor on some particular genes [30,31]. Nonetheless, the regulation of HP1γ on gene expression can result in transcriptional activation as well. A study by Eguchi et al. revealed that HP1γ was associated with the transcription of HSP70’B mRNA by cooperating with the hemopexin-like repeat domain of MMP3 protein and allows its DNA assembly at the promoter region [32]. Alternatively, it was found that HP1γ can promote the gene expression through inducing efficient RNA splicing [33]. The role of HP1γ in mediating efficient RNA processing can be related to the recruitment of splicing regulator SRSF1, which was found in the alternative splicing of VEGFA mRNA [34], without altering its mRNA expression. As both mRNA and protein levels of CDK1 and PCNA were responsible to CBX3 expression, it might be postulated that CBX3-encoding HP1γ may act as a co-operator of CDK1 and PCNA transcription factor to facilitate its promoter binding capacity and therefore promote gene transcription. The exact mechanism underlying the regulation of HP1γ on the expression of CDK1 and PCNA may need further exploration.

**Figure 7:**
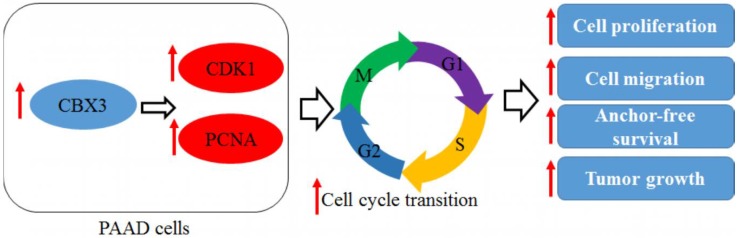
Overall regulatory mechanism involved in the action of CBX3 in PAAD. Red arrows indicated the changes of trend of protein expression and cellular activity when CBX3 is upregulated.

We observed that CDK1 may mediate the tumor-promoting role of CBX3 in PAAD (Figure 7). CDK1 is the determinant factor of mitotic entry in mammalian cells. It was concluded by knockdown experiment that CDK1 was required for the proliferation of mammalian cells as it was the only initiator of mitotic onset [35]. Moreover, in cells absent of other CDKs, CDK1 can execute all the events that are required for cell cycle division, indicating that CDK1 is sufficient for the proliferation of mammalian cells [36]. In PAAD, CDK1 overexpression was found in 54.8% of patients, and overexpression of CDK1 was directly related to lymph node metastasis and Ki-67 labeling index (LI) [37]. Inhibition of CDK1 activity can successfully suppress the in vitro and in vivo tumor growth of PAAD, further proving the pivotal role of CDK1 pathway in PAAD progression [38]. In our study, we found that knockdown of CDK1 induced the G2/M cell cycle arrest in CBX3-overexpressing cells, suggesting that the role of CBX3-induced CDK1 may be associated with the cell cycle transition of PAAD cells. Increased expression of CDK1 may recruit Cyclin B1 to form a protein complex that promotes anaphase transition and cell proliferation [39]. Furthermore, we found that knockdown of CDK1 may down-regulate the migration and invasion of CBX-overexpressing PAAD cells. Knockdown of CDK1 was found to defect cell migration in non-small cell lung cancer [40]. CDK1 may facilitate cell migration and invasion with multiple signaling pathways involved, including YAP [41] and HIF1α [42]. In pancreatic cancer, it was also observed that CDK1 can phosphorylate Vgll4, which in consequence activates the Hippo and Wnt pathways [43]. As Hippo and Wnt pathways may be involved in not only proliferation but also invasiveness of pancreatic cells, our observation as well as literature study suggests that CBX3-driven CDK1 expression may play an important role in the tumorigenesis of PAAD.

## 4. Materials and Methods

### 4.1. Chemicals, Antibodies, and Plasmids

The CDK1 inhibitor Ro-3306 and 4′,6-diamidino-2-phenylindole (DAPI) was purchased from Sigma-Aldrich Saint Louis, MO, USA). Antibodies against HP1γ, PCNA and CDK1 were obtained from Cell Signaling Technologies (Danvers, MA, USA). Control and CBX3 Crispr activation plasmids, scramble negative control, CBX3 siRNA and CDK1 siRNA were purchased from Santa Cruz Biotechnologies (Dallas, TX, USA).

### 4.2. Cells and Cell Culture

KP3L cells expressing luciferase reporter were purchased from JCRB cell bank (Tokyo, Japan). Cells were cultured in RPMI1640 medium supplemented with 10% fetal bovine serum (FBS) and 1% penicillin–streptomycin (Thermofisher, Waltham, MA, USA) in humidified condition with 5% CO_2_ at 37 °C. PANC-1 cells were purchased from American Type Culture Collection (ATCC, Manassas, VA, USA). Cells were maintained in DMEM medium supplemented with 10% fetal bovine serum (FBS) and 1% penicillin–streptomycin (Thermofisher) in humidified condition with 5% CO_2_ at 37 °C. 

### 4.3. Animal Study Protocol

Protocol of animal study was reviewed and approved by the ethic committee of the department of laboratory animal science, Fudan University (Ref. No. 20160673A040, Date of Approval: 18 February 2016). The orthotopic PAAD orthotopic murine model was established as previously described [44]. In brief, five-week-old female athymic nude mice were anesthetized with 100 mg/kg ketamine and 10 mg/kg xylazine. Then, 2 × 10^6^ KP3L cells mixed with Matrigel matrix (BD Bioscience, San Jose, CA, USA, 1:1 *v*/*v*) were injected into the tail of the pancreas via laparotomy. Each group contained 5 mice. Tumor growth was imaged under live animal imager (IVIS Spectrum, Perkin-Elmer, Waltham, MA, USA) once per week for three weeks. At the end of study, mice were sacrificed with overdose of pentobarbital (200 mg/kg, i.p.) and tumor-bearing pancreas was dissected out. Histological analysis using H&E staining was performed and images were captured under light microscope (400× magnification, Leica, Wetzlar, Germany).

### 4.4. Soft Agar Assay

Soft agar assay was performed to measure the anchorage-free growth of tumor cells. In brief, 1.5 mL culture medium containing 0.5% agar was added to 6-well tissue culture plate and set aside for 5 min to solidify the agar layer. Then, 1.5 mL culture medium containing 0.35% agar and 20,000 tumor cells was added to the top layer and left until the top layer was solid. Two milliliters of culture medium were then added and the plate was cultured at 37 °C for 14 days. At the end of the experiment, cells were stained with 0.005% crystal violet for 1 h and images were captured under Chemidoc Imaging system (bio-rad, Hercules, CA, USA). Number of colony formed was counted.

### 4.5. Wound Healing and Invasion Chamber Assay

For measuring cell migration, KP3L and PANC-1 cells were cultured in 6-well plate until full coverage. A wound was created by scraping cell layer using a 10 μL pipet tip. Images were captured at 0 and 48 h to observe the wound closure by cell migration. For measuring cell invasion, 1 × 10^5^ KP3L and PANC-1 in serum-free medium was seeded onto the chamber insert (Corning, Corning, NY, USA) coated with Matrigel Matrix (1:3 *v*/*v* in cold PBS, BD Bioscience). The received chamber was filled with medium containing FBS. Forty-eight hours after cell seeding, cells attached at the basolateral membrane of chamber insert were fixed and stained with crystal violet. Images were captured under microscope (200× magnification, Leica).

### 4.6. Quantitative Real-Time PCR

Total RNA was extracted with Trizol reagent (Thermofisher) and first strand cDNA was synthesized (Roche, Basel, Switzerland). Quantitative real-time PCR was performed with SYBR master mix (Roche, USA) on the platform of LC480 (Roche). Primers used were as follows, CBX3: (Forward) 5′-GGTGAACTCTTCAAGTCTCCG-3′, (Reverse) 5′-TTATTGTGCT TCATCTTCAGGACAAG-3′; PCNA (Forward) 5′-GGCTCCATCCTCAAGAAGGT-3′, (Reverse) 5′-AGTCCATGCTCTGCAGGTTT-3′; CDK1 (Forward) 5′-TTTTCAGAGCTTTGGGCACT-3′, (Reverse) 5′-CCATTTTGCCAGAAATTCGT-3′; GAPDH (Forward) 5′-CATGAGAAGTATGACAACAGCCT-3′, (Reverse) 5′-AGTCCTTCCACGATACCAAAGT-3′. Expression of CBX3, PCNA and CDK1 was normalized by GAPDH.

### 4.7. Immunoblotting

Total protein was extracted with RIPA buffer (Sigma-Aldrich). Protein concentration was determined by BCA method (Bio-rad) and same amount of total protein was loaded to sodium dodecyl sulfate polyacrylamide gel electrophoresis (SDS-PAGE) for electrophoresis. Protein was then transferred to PVDF membrane (Roche), followed by antigen blocking in TBST buffer containing 5% BSA (Sigma-Aldrich) for 2 h. Primary antibody incubation was performed overnight at 4 °C, followed by appropriate secondary antibody incubation for 2 h at room temperature. Blot was imaged with ECL select (GE Healthcare, Chicago, IL, USA) as substrate in Chemidoc imaging system. Intensity of the target bands were quantified by Image J and normalized by β-actin.

### 4.8. Quantification of Mitotic Index

Quantification of mitotic index was performed according to previous publication [45]. In brief, cells were fixed with cold methanol/acetone (1:1, *v*/*v*) for 10 min followed by DAPI staining for 20 min in dark. Cells were observed under fluorescence microscope and cells with condense nuclear DNA were considered to undergo mitosis. Percentage of mitotic cells out of total viable cells were calculated as mitotic index and data was collected from three independent experiments.

### 4.9. Statistical Analysis

Comparison was performed with student *t*-test and *p* < 0.05 was considered statistically significant.

## 5. Conclusions

In this study, we identified the pattern, function and regulation of CBX3 in human PAAD. We found that CBX3 mRNA and its encoding protein HP1γ were overexpressed in human PAAD. Overexpression of CBX3 in PAAD patients was negatively correlated with the overall and progression-free survival of patients. In PAAD cells, overexpression of CBX3 induced cell proliferation, migration and invasion, and promoted the in vivo tumor growth in nude mice. GO and KEGG analysis identified that the action of CBX3 in PAAD may involve cell cycle regulation, and cell cycle protein CDK1 and PCNA may be the down-stream target of CBX3. Expression of CDK1 and PCNA in PAAD positively correlated with CBX3. CBX3 promoted cell cycle transition from G2/M checkpoint. Knockdown of CDK1 in CBX3-overexpressing PAAD cells accumulated cells at G2/M phase, which led to reduced cell proliferation, migration and invasion of CBX3-overexpressing PAAD cells. These findings suggest the tumor-promoting role of CBX3 in PAAD and novel therapeutic approaches targeting CBX3 may deserve consideration.
